# Recrystallization as the governing mechanism of ion track formation

**DOI:** 10.1038/s41598-019-40239-9

**Published:** 2019-03-07

**Authors:** R. A. Rymzhanov, N. Medvedev, J. H. O’Connell, A. Janse van Vuuren, V. A. Skuratov, A. E. Volkov

**Affiliations:** 1 Joint Institute for Nuclear Research, Joliot-Curie 6, 141980 Dubna, Moscow Region Russia; 2 The Institute of Nuclear Physics, Ibragimov St. 1, 050032 Almaty, Kazakhstan; 3 L. N. Gumilyov Eurasian National University, Satpayev St. 2, 010008 Astana, Kazakhstan; 4 Institute of Physics, Czech Academy of Sciences, Na Slovance 2, 182 21 Prague 8, Czech Republic; 5 Institute of Plasma Physics, Czech Academy of Sciences, Za Slovankou 3, 182 00 Prague 8, Czech Republic; 6 Nelson Mandela University, University way, Summerstrand, 6001 Port Elizabeth, South Africa; 7 National Research Nuclear University MEPhI, Kashirskoye sh., 31, 115409 Moscow, Russia; 8 Dubna State University, Universitetskay 19, 141980 Dubna, Moscow Region Russia; 9 National Research Center ‘Kurchatov Institute’, Kurchatov Sq. 1, 123182 Moscow, Russia; 10 Lebedev Physical Institute of the Russian Academy of Sciences, Leninskij pr., 53, 119991 Moscow, Russia; 11 National University of Science and Technology MISiS, Leninskij pr., 4, 119049 Moscow, Russia

## Abstract

Response of dielectric crystals: MgO, Al_2_O_3_ and Y_3_Al_5_O_12_ (YAG) to irradiation with 167 MeV Xe ions decelerating in the electronic stopping regime is studied. Comprehensive simulations demonstrated that despite similar ion energy losses and the initial excitation kinetics of the electronic systems and lattices, significant differences occur among final structures of ion tracks in these materials, supported by experiments. No ion tracks appeared in MgO, whereas discontinuous distorted crystalline tracks of ~2 nm in diameter were observed in Al_2_O_3_ and continuous amorphous tracks were detected in YAG. These track structures in Al_2_O_3_ and YAG were confirmed by high resolution TEM data. The simulations enabled us to identify recrystallization as the dominant mechanism governing formation of detected tracks in these oxides. We analyzed effects of the viscosity in molten state, lattice structure and difference in the kinetics of metallic and oxygen sublattices at the crystallization surface on damage recovery in tracks.

## Introduction

A swift heavy ion (SHI, Е >1 MeV/nucl) loses the largest part of its energy (>95%, 5–50 keV/nm along the ion trajectory) through excitation of the electronic subsystem of a solid^1,2^. Subsequent relaxation of the perturbation occurs at ulrashort spatial (nanometers) and temporal (femto- to pico-seconds) scales and cannot be described in the framework of usual macroscopic models^3,4^. As a result, unusual kinetics often produce unusual structure and phase transformations which constitute a latent ion track: structure modified material with a diameter of ~1–10 nm and a length of ~10–100 μm along the trajectory of a projectile. The identification of mechanisms governing these kinetics forms a fundamental field of interest in SHI track effects.

Various nanometric features created along an ion path form technological interest in SHI irradiations^5^. Material transformations in ion tracks motivate applications of SHI irradiations for nanostructuring^2,6–8^. Created tracks form the basis for further manufacturing of nanowires^9^ and track membranes^10,11^. SHIs are used for fabrication of quantum devices^12,13^, ion therapy of oncological diseases^14^, etc. Additionally, shielding from the SHIs produced as fission fragments in nuclear fuel relies on the resistance of materials to SHI irradiation in order to safely handle such fuel^15^. For all of these applications, a choice of the most suitable materials traditionally had to be made mainly by trial and error. Understanding of the governing mechanisms of the track formation will enable control and design of the nanoscale patterns in materials with desired qualities.

SHI penetration trough different insulators have shown quite different manifestations of structure transformations^1,8^: amorphous tracks (e.g. Y_3_Fe_5_O_12_, α-quartz), crystalline tracks (e.g. Mg_2_AlO_4_, Al_2_O_3_) or production of isolated point defects and color centers (e.g. alkali halides). This motivates research aimed at the understanding of mechanisms of track formation in these materials.

Track formation is a typical example of multiscale physics. It consists of well separable stages: excitation (at attosecond timescales) and relaxation (femtoseconds) of the electronic subsystem of a target, excitation of the lattice due to energy transfer from electrons (picoseconds), followed by structure changes during lattice relaxation (up to nanoseconds). In turn, the kinetics of structure transformations can be divided into: (a) formation of an initial damage around the ion trajectory, and (b) relaxation of this initial damage into a final damaged structure^16^. As with most of the multiscale physics problems, track formation cannot be traced accurately within a single model posing formidable challenges for theoretical description and understanding of the underlying physical mechanisms.

In order to study, which of the above-mentioned process is the main governing mechanism of track formation, we have chosen three dielectric materials (Al_2_O_3_, MgO and Y_3_Al_5_O_12_) with different lattice structures but comparable energy deposited into the lattice. It was shown that SHI irradiation of these targets leads to formation of quite different damaged structures, despite the fact that the initial damaged regions have similar sizes and structures. This allows us to study the fundamental effect of influence of lattice structure on the relaxation kinetics of an exited solid and reveal mechanisms of recrystallization in these oxides.

## Experiment

We compared the responses of single crystalline α-Al_2_O_3_, MgO and Y_3_Al_5_O_12_ (YAG) specimens to irradiation with 167 MeV Xe ions at 300 K. The irradiations were performed at fluences ranging from 10^10^ to 10^12^ cm^−2^ at the IC-100 cyclotron at FLNR JINR (Dubna, Russia) in order to avoid track overlap; average Xe ion flux was ~10^9^ cm^−2^ s^−1^. Ion beam homogeneity better than 5% at the surface of an irradiated specimen has been reached using beam scanning in horizontal and vertical directions. High resolution transmission electron microscopy (HRTEM) studies were carried out at the Centre for HRTEM at Nelson Mandela University (Port Elizabeth, South Africa). Plan view TEM lamellae were extracted within 1 µm from the irradiated surface using an FEI Helios Nanolab 650 FIB. Focused 30 keV Ga ion beam was used for milling and pre-thinning of samples, while 1 keV Ga ions were used for final polishing. Samples were imaged using JEOL ARM-200F TEM operating at 200 kV, achieving higher resolution than in the previous TEM analysis^16^.

## Model

A hybrid approach used in this work consists of two models: Monte Carlo simulation (MC code TREKIS^17,18^) of the electron kinetics, and Molecular Dynamics (MD) model of atomic dynamics. TREKIS^17,18^ describes temporal evolution of excited electrons generated by an SHI as well as interaction of primary and secondary electrons with target lattice in an ion track. The resulting distribution of energy transferred to an ionic subsystem of a target is implemented into classical MD code LAMMPS^19^ which is used to simulate lattice energy relaxation and further structure transformations in the closest vicinity of the ion trajectory. No *a-posteriori* fitting parameters are used in the model^16^.

The event-by-event Monte-Carlo simulation of propagation of charged particles forms the basis of the MC approach^20–22^. TREKIS models the following processes^17,18^: (a) passage of a swift heavy ion through the solid ionizing the target and generating δ-electrons and holes; (b) propagation of primary and secondary electrons and their scattering on target atoms and electrons; (c) spatial redistribution of holes in the valence band and their interaction with collective atomic and electronic modes of a target; (d) secondary electrons generations via Auger decays of deep shell holes; (e) decays of deep shell holes through emission of photons, as well as further transport of light and its absorption producing new secondary electrons.

The cross sections used in TREKIS take into account collective responses of the electronic and the atomic systems of a target in the framework of the dynamic structure factor (DSF) and complex dielectric function (CDF, $\varepsilon (\omega ,q)$) formalism providing an adequate description of the track kinetics without any fitting. E.g., in the recent works such methods achieved outstanding precision^23^. The following form of the differential cross section of a charged particle interaction with a solid is used^24^:

1 $$\frac{{d}^{2}\sigma }{d(\hslash \omega )d(\hslash q)}=\frac{2{({Z}_{e}(v,q)e)}^{2}}{{n}_{sc}\pi {\hslash }^{2}{v}^{2}}\frac{1}{\hslash q}(1-{e}^{\frac{-\hslash \omega }{{k}_{B}T}}{)}^{-1}{\rm{I}}{\rm{m}}\,[\frac{-1}{\varepsilon (\omega ,q)}]$$

where *Z*_*e*_(*v*, *q*) is the effective charge of the projectile penetrating through a solid as a function of its velocity, *v*, and transferred momentum, **q** (for an incident electron *Z*_*e*_ = 1, for an SHI see the discussion about *Z*_*e*_ in ref.^17^); *ħω* is the transferred energy in the considered scattering event; *e* is the electron charge; *ħ* is the Planck constant; *k*_*B*_ is the Boltzmann constant, and *T* is the temperature of the sample; *n*_*sc*_ is the density of scattering centers.

The CDF can be reconstructed from optical data (refractive index and extinction coefficient, or a photon attenuation length). To extract the analytical form of the inverse imaginary part of the CDF, the dependence of the loss function on the transferred energy is then approximated by a set of artificial optical oscillators^25^. The numerical details and the reconstructed loss functions for different interaction channels in MgO and Al_2_O_3_ can be found in refs^17,18^; cross sections parameters for YAG are presented in Supplementary Materials.

The calculated cross sections are implemented into asymptotic trajectory Monte Carlo code (TREKIS^17^) using the Poisson distribution for the free-flight distance^22,24^ and the mean free path of a projectile scattering. During penetration of a charged particle, the target is assumed to be homogeneous atom and electron arrangements with the densities corresponding to the solid densities of the materials, and no orientation effects are taken into account, such as channeling of the SHI. No defects or impurities in the material are included in these MC simulations. Target electrons are considered as uniformly distributed particles occupying either the deep atomic energy levels^26^ or the states in the valence or conduction bands according to the density of states (DOS) of a material. Taking into account large velocities of projectiles, we assume these electrons as point-like particles at fixed positions during their energy and momentum exchange with an SHI (instant collisions).

After ~10^3^ iterations of the MC procedure and statistical averaging of the results^17,18^ we obtain the spatial (cylindrical geometry) and temporal distributions of the densities and energies of electrons, valence and core holes, as well as the density of energy deposited into the target lattice by electrons and holes^16^. Initial excitation of atomic subsystem in a SHI track is attributed to two sources: (1) Elastic scatterings of generated electrons and valence holes on optical phonons resulting in energy transfer to target lattice; (2) Release of potential energy into the electronic subsystem via electron-hole recombination, such as three-body recombination, allowing for further heating of the target atoms. An instant transfer of the potential energy stored in valence holes into the atomic subsystem at time 100 fs after SHI passage is assumed. In ref.^16^, we illustrated validity of our method.

Using the MC calculated distribution of the initial energy transferred into the lattice, the atom velocities were set in cylindrical layers around the SHI trajectory. Within each cylindrical layer, the distribution of the kinetic energy assumes a Gaussian dispersion of velocity modulus and a uniform momentum direction of atoms^16^. These velocities distributions are used as input data for MD code LAMMPS^19^ to model subsequent lattice relaxation and final structure modifications. Interatomic forces in YAG are simulated with the three-body interaction potential developed in ref.^27^, whereas the interaction between atoms in Al_2_O_3_ and MgO is described with pair Buckingham-type potentials with parameterization taken from^28^. YAG potential was thoroughly tested in the ref.^27^, where thermodynamical properties (melting point, heat conductivity and capacity) were studied in detail. Additionally, we have tested these potentials determining melting points of the studied materials: T_melt_(Al_2_O_3_) = 2250 K (2345 K), T_melt_(MgO) = 2560 K (3125 K), T_melt_(YAG) = 3300 K (2213 K); experimental values are given in brackets. We also calculated elastic constants for Al_2_O_3_ and MgO which are in reasonable agreement with experimental values. In case of Al_2_O_3_, a good reproduction of SHI induced effects in this material in a wide range of irradiation regimes studied in our previous work^16^ also confirms applicability of this interatomic potential, serving as a benchmark calculation.

The supercell sizes constructed for the MD modeling were 24.8 × 24.8 × 14.5 nm^3^ for MgO (1008000 atoms), 20.4 × 20.5 × 19.4 nm^3^ for Al_2_O_3_ (967500 atoms) and 25.9 × 25.9 × 16.1 nm^3^ for YAG (917280 atoms)  with periodic boundary conditions in all directions (bulk simulation). Ion trajectories were parallel to Z direction, while the temperature of borders of the supercell perpendicular to the X- and Y-axis is maintained by the Berendsen thermostat^29^ at 300 K with the damp time of 100 fs. The kinetics of target lattice relaxation is followed up to 150 ps, after which the whole supercell is cooled down to <400 K, so after this time we do not expect any structural changes.

Visualization of the states of MD cells at different times after impact of high energy heavy ion is performed using OVITO open-source software^30^.

## Results

**Figure 1:**
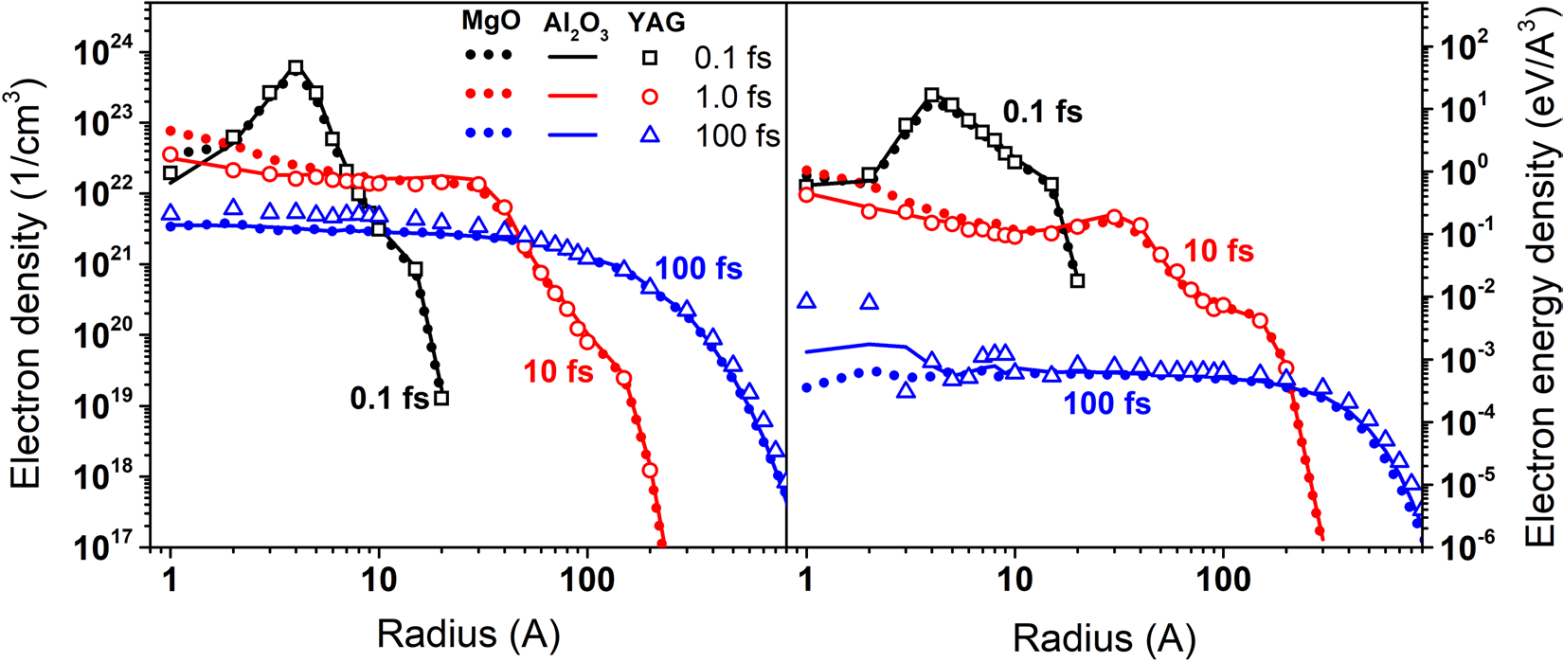
Radial electron density (left panel) and energy density (right panel) in MgO (filled circles), Al_2_O_3_ (solid lines) and YAG (open symbols) at different times after 167 MeV Xe ion passage. Color coding indicates different time instants.

The calculated energy loss of a 167 MeV xenon ion in MgO, Al_2_O_3_ and YAG are 21, 24.9 and 25 keV/nm respectively. Figure 1 shows the radial and temporal dependencies of the energy of electrons and their density after 167 MeV Xe impact onto these three solids. One can see that the kinetics of the electron subsystem differ only slightly among MgO, Al_2_O_3_ and YAG, besides electron energies at small radii (<1 nm).

**Figure 2:**
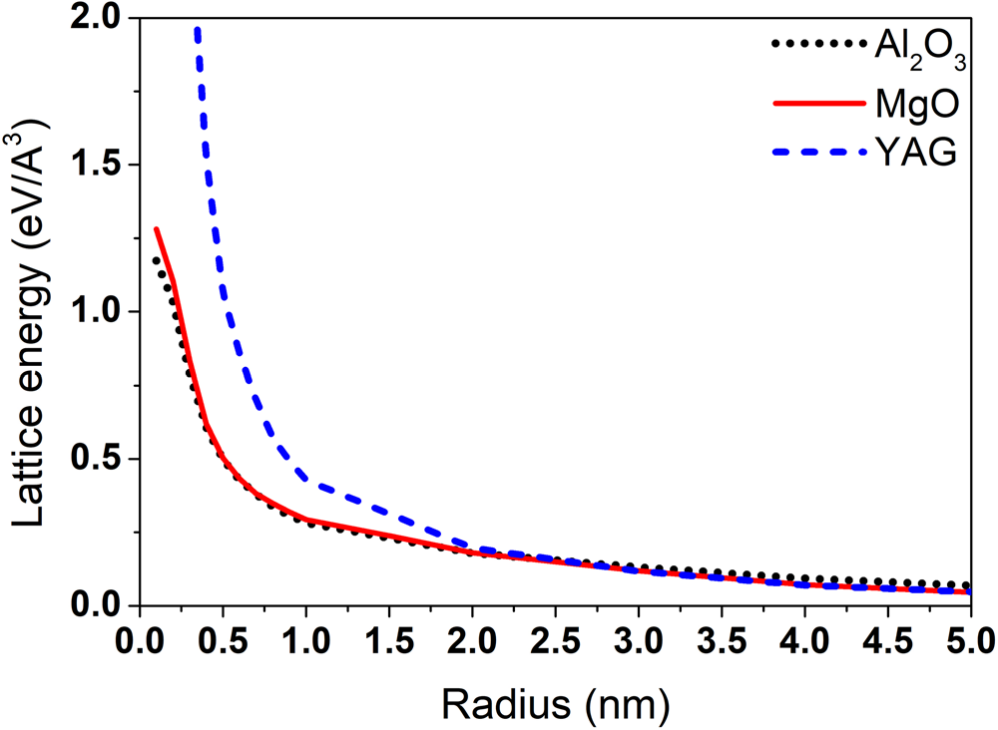
Radial distribution of the excess lattice energy density around the trajectories of 167 MeV Xe in MgO, Al_2_O_3_ and YAG.

After the electron kinetics, the radial distributions of the energy transferred into the lattices of MgO, Al_2_O_3_ and YAG are presented in Fig. 2. All these three targets demonstrate similar behavior in kinetics of electronic excitation and relaxation: formation of two fronts of excess energy propagation and comparable energy deposition to the lattice (a slightly higher energy density in YAG within ~1 nm around the ion path introduces only a negligible difference, since the volume of this region is very small). Nearly identical kinetics of excitation allows us to use these three systems to isolate an effect of lattice relaxation on the track formation in oxides. The study of lattice relaxation processes can help to understand the influence of a structure and a force field of atoms, as will be discussed below.

**Figure 3:**
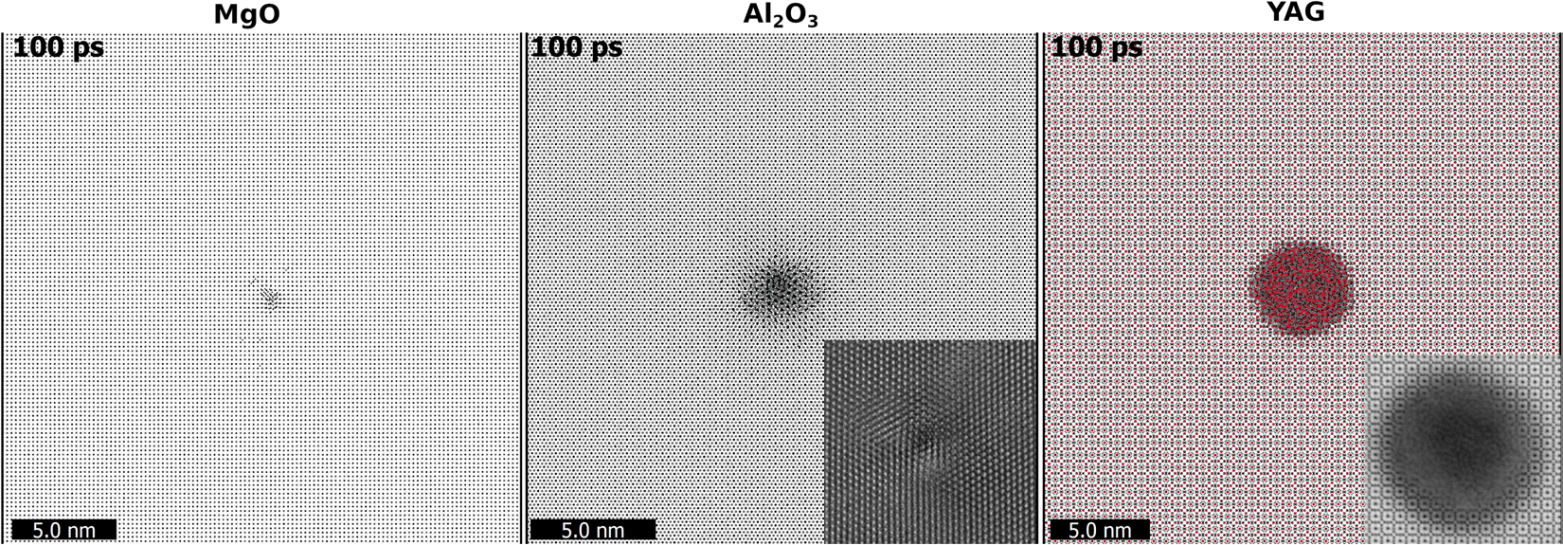
Snapshots of modeled 167 MeV Xe tracks in three materials at 100 fs after ion passage with the experimental latent tracks as insets. The scales of MD images and TEM insets are the same.

Despite comparable energy deposition into the lattice (especially for radii >1 nm), the passage of 167 MeV Xe produced notably different damaged structures in the investigated oxides (Fig. 3).

MD supercell of MgO contains only a few point defects in the proximity of the ion trajectory with no SHI track after lattice relaxation (similar situation occurs for 700 MeV Bi irradiation). The absence of SHI damage in MgO was also detected in experiments^31^ and hence no TEM imaged track is shown for this material in Fig. 3.

The simulation predicted that 167 MeV Xe in Al_2_O_3_ forms a discontinuous crystalline track of ~2 ± 0.3 nm in diameter, which was confirmed by high resolution TEM images and was discussed in detail in our previous works^16^. Although a part of the experimental results was published before, here the experimental samples were reanalyzed with a high resolution TEM for better comparison with the model predictions. Typical FIB lamellae have thickness around 30–40 nm and thus such small localized areas of distorted crystal are always imaged within the relatively pristine matrix. An HRTEM micrograph of such a track along the [0001] crystallographic direction is shown as an inset at the same scale as the Al_2_O_3_ simulation in Fig. 3. The track core region can be distinguished from the surrounding matrix near the center of the inset. Strains around the track periphery are visible as small localized deviations from the perfect zone axis alignment leading to discrete bright atomic columns merging into lines of bright contrast.

A calculated track in YAG has a cylindrical shape with the size of ~5.1 ± 0.2 nm and a completely amorphous structure, in a reasonable agreement with experiment, Fig. 3. The experiment confirms that the tracks in YAG are amorphous with a comparable diameter of ~6.5 ± 0.6 nm based on 97 measured tracks. The inset shows an annular bright field (ABF) HRSTEM micrograph of a typical track in YAG. In this imaging mode, the dark central region with a relatively flat contrast represents an amorphous track core. The amorphous-crystalline boundary can be seen just at the edge of the dark circle where atomic columns become visible. The strain field within the crystalline region causes further darkening around the track periphery.

The good coincidence with the experimental results confirms the applicability of the presented model and used interatomic potentials for description of the track kinetics in these oxides. The cross-sectional MD images of tracks in these solids are shown in Supplementary Materials.

**Figure 4:**
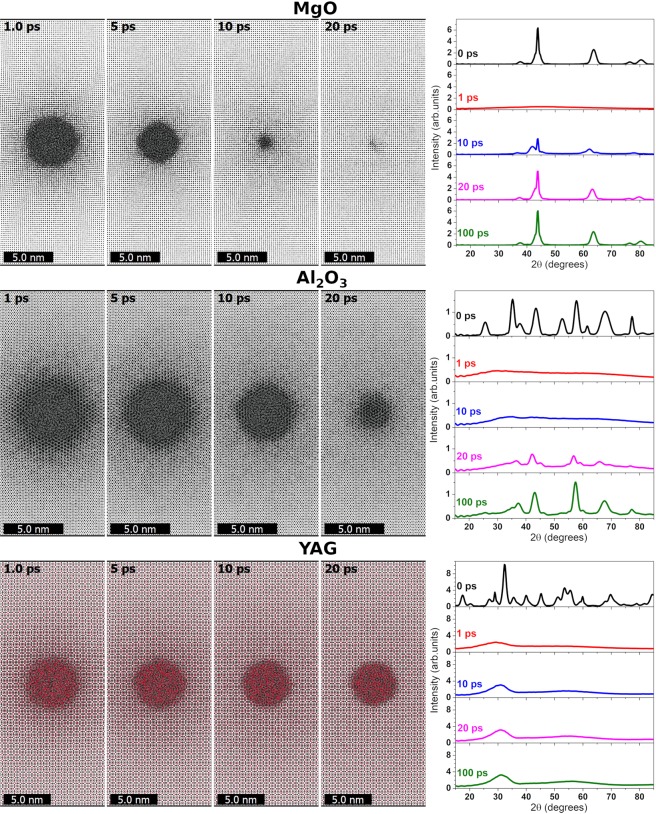
Snapshots of MD supercell (left panels) and simulated X-ray powder diffraction patterns (right panels) of MgO, Al_2_O_3_ and YAG at different times after the passage of 167 MeV Xe ion.

Considering the structure kinetics in a track by means of MD, we observe that the initial states of the perturbed lattice (at ~1 ps) are very similar for all three samples: a disordered area of almost the same diameter (~5–6 nm), see Fig. 4. We calculated X-ray powder diffraction patterns for the cylinder of 4 nm around the ion path with the help of Debyer code^32^ (finite width of the peaks is due to the finite size of the cylinder). Examination of these patterns reveals that an amorphous structure of the damaged area is present during the early times (<5 ps, right panels of Fig. 4) i.e. transient material melting occurs around the ion trajectory for all the considered materials.

Summarizing, Figs 1, 2 and 4 demonstrate that the initial electronic kinetics, energy transfer to the lattice, and lattice melting in the nearest vicinity of the projectile trajectory, are nearly identical for these three targets. But Figs 3 and 4 also show that structure transformations during further cooling of the melted cylinders strongly differ in MgO, Al_2_O_3_ and YAG (snapshots for times ≥5 ps in Fig. 4).

In the case of MgO, the amorphous region recrystallizes epitaxially to an almost virgin state fast (by the time of ~20 ps). At 100 ps, the powder diffraction patterns are almost the same as for the undamaged material. Only a small number of point defects are still present in the closest vicinity of the SHI trajectory. The recovery of initial damage in alumina is slower and finally results in formation of a small crystalline track. The diffraction patterns show that the material recovers its structure only partially. The size of a disordered region in YAG reduces only slightly during cooling, forming finally an amorphous track, as also confirmed by the diffraction pattern.

## Discussion

Despite the great interest for fundamental understanding of material behavior under extreme levels of excitation, the role of recrystallization of solids in tracks of swift heavy ions is still an open question. In this work, we used a combined approach consisting of the MC simulations of a target excitation with MD model of atomic kinetics. It provided us with energy distributions in a SHI irradiated area without any unnecessary and unjustified assumptions influencing the excitation stage of track production and the final size of an ion track.

Applying this approach to various combinations of ions and dielectric targets, we choose three oxides with different crystalline structure where the same SHIs produce similar initial excitations of the lattice. This allowed us to decouple different processes in SHI tracks and to investigate specifically effects of relaxation of the lattice on track formation in these materials. The experiments as well as simulations of the relaxation kinetics of excited tracks in these oxides demonstrated their different reaction resulted finally in very different structure transformations. This indicates that excitation is a subdominant process in comparison to recrystallization in the kinetics of SHI track formation in these oxides.

**Figure 5:**
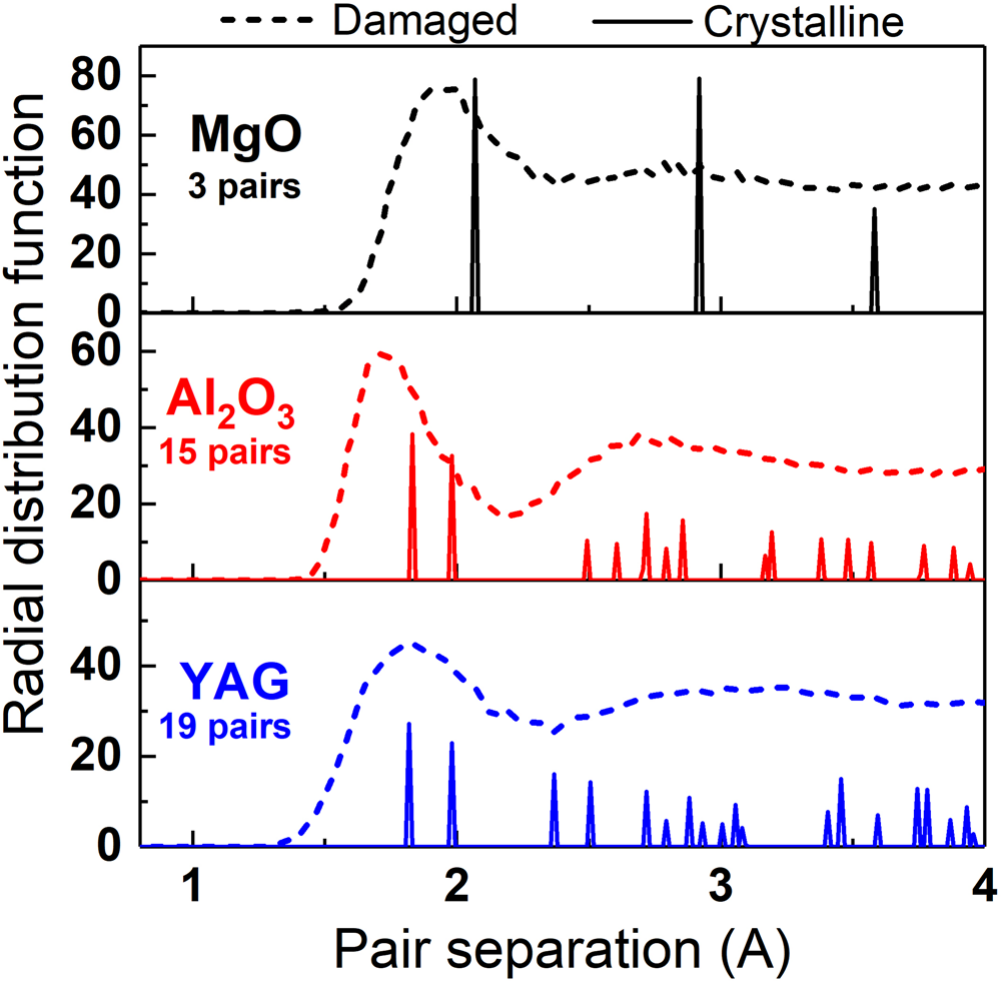
Modeled radial distribution functions of MgO, Al_2_O_3_ and YAG in the virgin states (solid lines) and at 1 ps after 167 MeV Xe impact (dashed lines).

First, we checked whether there is any ordering effect of bonds between atoms at the initial stage of track formation, which can enhance or suppress recrystallization. The analysis of pair distribution functions of studied materials does not reveal any considerable difference between MgO, Al_2_O_3_ and YAG at initial moments (at ~1 ps after an ion impact) of relaxation of excited tracks (see Fig. 5). Thus we can conclude that all materials transiently lose their structure, and recrystallization starts from a similar lattice with initial short order states in these oxides.

One can also note that the recrystallization efficiency is in correlation with “simplicity” (or “complexity”) of the target lattice structure, as it was suggested in e.g. ref.^1^. To quantify these terms, in the simplest case we can define “complexity” here as a number of pairs of atoms within a characteristic interatomic distance. This number can be represented by the number of peaks in the static lattice pair correlation function which are given in Fig. 5. Indeed, complete and fast track recrystallization is observed in MgO having a “simple” lattice structure with the smallest number of the peaks in the pair correlation function. A similar picture was observed in MD modeling of LiF and NaCl exhibiting no continuous tracks^33^. But large amorphous tracks are detected in YAG with the most “complex” lattice structure (the largest number of peaks). Reference^34^ showed that SHI irradiation of Mg_2_SiO_4_ with a “complex” structure produces amorphous tracks which conforms to the presented trend. The lattice of Al_2_O_3_ fills an intermediate “complexity” here, showing partial track formation. The observed correlation allows us to conclude that the “complexity” of the structure can be used for a crude estimate of the expected recrystallization efficiency in SHI tracks. However, the complexity of a lattice structure cannot be treated as the sole factor governing damage formation in SHI tracks, but should be augmented in more general considerations, involving dynamical properties of materials.

Epitaxial recrystallization is a process of atoms moving from a molten region to their equilibrium positions at the interface with the crystalline structure. We observed that an ability of amorphization of SHI tracks in these oxides are well correlated with the viscosity in the molten state, which is related to effects of fluidity dynamics of stressed melts as was known in the literature (see e.g. ref.^35^ and references therein). We estimated viscosity of liquid MgO, Al_2_O_3_ and YAG at temperatures of 100 K above the melting point using molecular dynamics and Green-Kubo formalism (see Table 1)^36,37^. As expected, materials with higher viscosity exhibit larger tracks.

**Table 1:** Viscosities in the studied materials in their molten states at temperatures of T_melt_ + 100 K.

Material	Viscosity, mPa·s
MgO	1.44
Al_2_O_3_	4.97
Mg_2_SiO_4_	6.13
YAG	9.58

**Figure 6:**
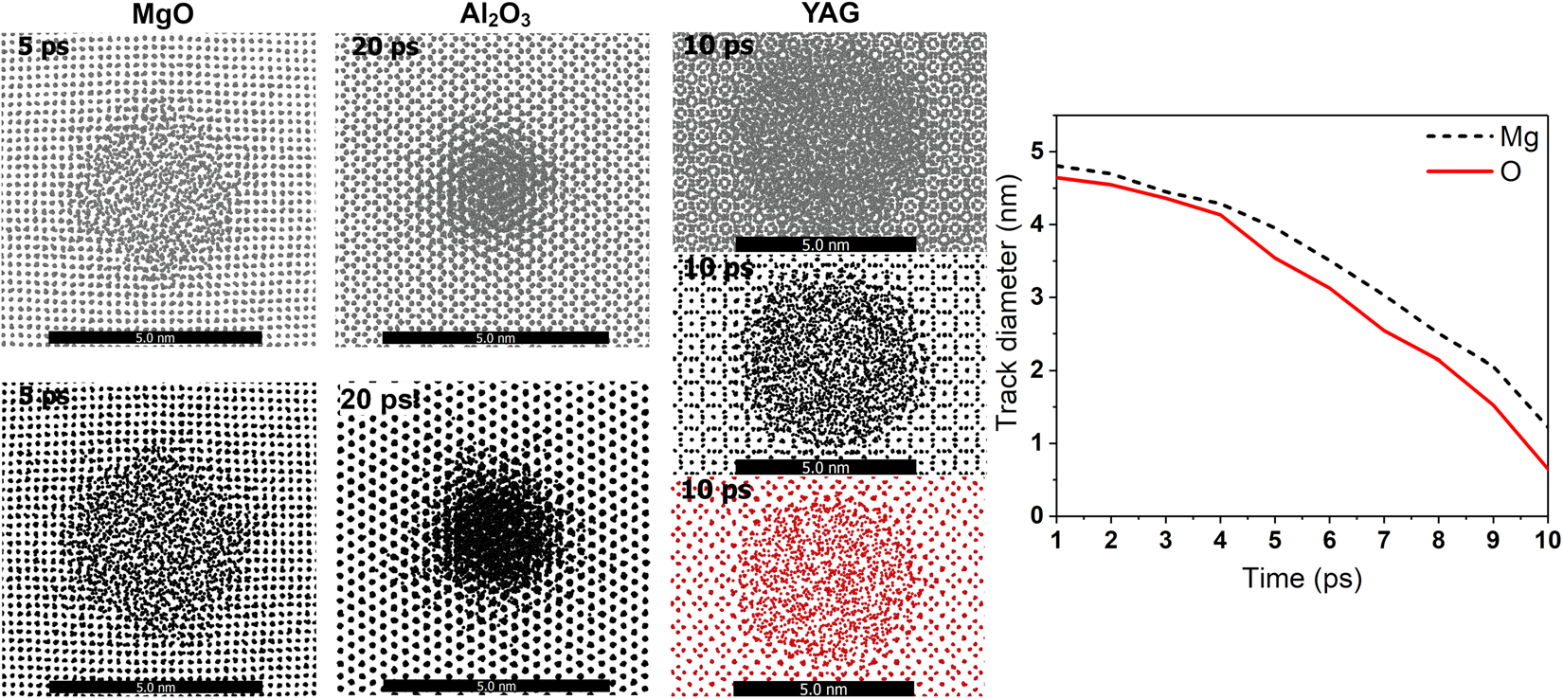
Left panel, snapshots of MD cells of MgO, Al_2_O_3_ and YAG sublaticces after passage of 167 MeV Xe ion. Time instances show the initial stage of track size reduction (recrystallization). Grey dots are oxygen, black dots are Mg (MgO) and Al (Al_2_O_3_, YAG), red points are Y atoms. Right panel shows evolution of damaged track diameters in oxygen and magnesium sublattises of MgO.

However, such a macroscopic quantity as viscosity does not allow to identify microscopic effects governing the recrystallization. In order to study the dynamics of the recrystallization in more details, we have considered the relaxation of sublattices, which revealed that oxygen atoms settle in their sites at epitaxially restoring interface of the crystalline matrix faster than Mg (or Al) atoms in MgO (Al_2_O_3_). Metallic atoms then adjust to a layer of oxygen that has already been built. Figure 6 shows temporal evolution of the sublattices of the studied targets at different times after the ion passage. Figure 6 (left) demonstrate that a diameter of the damaged region in the oxygen sublattice is smaller than in Mg (Al) sublattices in the case of MgO (Al_2_O_3_) during the relaxation stage.

The importance of oxygen kinetics was also mentioned in the works of Zhang *et al*.^38^ and Sachan *et al*.^39^. It was shown that pyrochlore Gd_2_Zr_2_O_7_ strongly recrystallizes after SHI passage, whereas Gd_2_Ti_2_O_7_ having the same structure produces amorphous tracks. Moreover, the intermediate structures Gd_2_Ti_x_Zr_(1−x)_O_7_ demonstrate the decrease of recrystallization efficiency with the increase of Ti content^39^. This indicates, in fact, that bonding strength of oxygen atoms has a significant effect on the damage recovery in these solids in SHI tracks. Indeed, the structures of the oxygen sublattices in Gd_2_Zr_2_O_7_ and Gd_2_Ti_2_O_7_ are similar, but there is a difference between Zr-O and Ti-O interaction potentials in these materials^38^. Ti-O pairs have a stronger bonding (potential energy minimum is −38.8 eV) than that of Zr-O bonds (−34.8 eV). This can suppress recrystallization in the oxygen sublattice by reducing oxygen migration velocity as mentioned in^38^.

**Figure 7:**
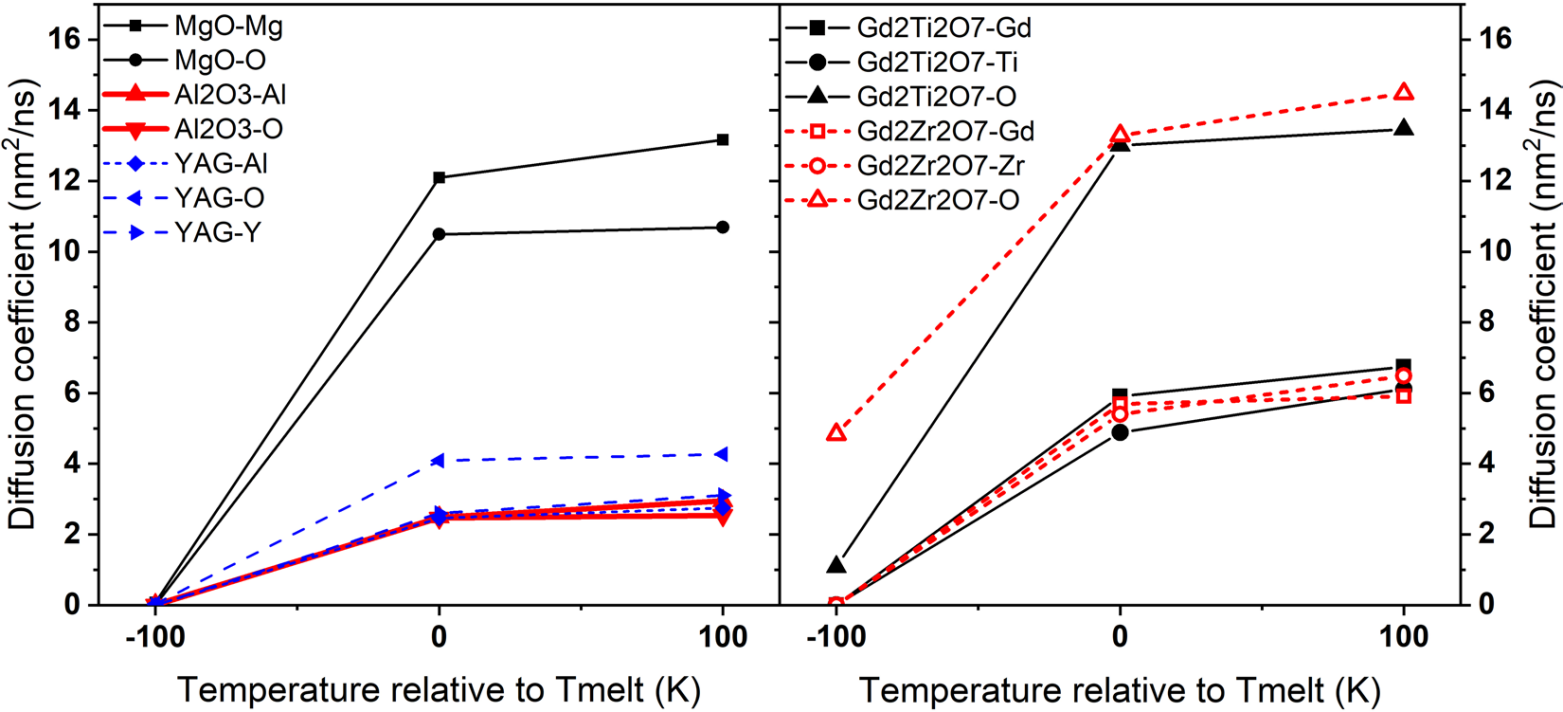
Diffusion coefficients of atoms in MgO, Al_2_O_3_ and YAG (left panel) and of Gd_2_Zr_2_O_7_ and Gd_2_Ti_2_O_7_ (right panel).

For an illustration of the effect of atomic mobility, we simulated the diffusion coefficients of atoms in studied oxides to check their importance in the track formation kinetics (Fig. 7). Since we are interested in the behavior of atoms near the interface between the transiently damaged (melted) region and the surrounding crystalline matrix, the migration velocities of atoms were studied at the melting temperature as well as at temperatures ±100 K from the melting point. The melting temperatures were calculated for each particular interatomic potential used for the studied materials. The diffusion coefficients were determined as the slopes of mean-squared displacements versus time.

Atoms in MgO demonstrate the highest diffusion coefficients in the molten regime, while the diffusion coefficient of oxygen in Al_2_O_3_ does not differ from that in YAG. Below the melting points, all three targets show almost zero diffusivity. Gd_2_Zr_2_O_7_ has a higher migration velocity at sub-melting temperatures than Gd_2_Ti_2_O_7_ and this difference strongly increases with decrease of temperature, which is consistent with the results presented in^38^.

It is interesting to note that the interatomic potentials used in MD produce the highest melting temperature in YAG among the studied oxides. However, despite an earlier onset of relaxation of a melted SHI track in YAG, there is not enough time to restore the crystalline structure. This shows that the difference in the recrystallization processes in SHI tracks cannot be attributed to differences in their melting points (as suggested e.g. in^38^).

Considering all the factors listed above, we conclude that the possibility of oxygen atoms to reach their equilibrium crystalline sites govern recrystallization of SHI tracks in the presented materials. Fast diffusion of oxygen facilitates occupation of the crystalline sites by these atoms during solidification of the melted tracks in MgO. In contrast, slower diffusion of oxygen atoms to their equilibrium positions during cooling of the nanometric molten region decreases a probability of track recrystallization in Al_2_O_3_ and YAG. We also saw that having the same diffusion coefficients as in YAG, oxygen atoms occupy their equilibrium positions faster on a “simpler” structure of Al_2_O_3_. This results in formation of discontinuous crystalline tracks in alumina in contrast to appearance of amorphous tracks in YAG. The structures in Gd_2_Zr_2_O_7_ and Gd_2_Ti_2_O_7_ are similar, so these targets should have no significant difference in oxygen path lengths to its ideal position. In this case, the diffusion velocity of oxygen atoms becomes the governing factor of recrystallization of SHI tracks in Gd_2_Zr_2_O_7_ and Gd_2_Ti_2_O_7_.

## Conclusions

In conclusion, we observed both, experimentally and theoretically, that notably different ion tracks were produced in three oxides: MgO, Al_2_O_3_, and YAG irradiated with Xe (176 MeV) ions. In MgO, only point defects were created around the ion trajectory, with no track *per se*. In Al_2_O_3_ a crystalline discontinuous track with the diameter about 2 nm was formed. In YAG, a continuous amorphous track of ~6.5 nm in diameter was observed.

Detailed modeling with MC-MD hybrid code revealed that the initial electronic excitations in these materials are almost the same. The energy transferred from an excited electronic system to atoms is also nearly identical. It results in transient melting in the three materials within the same radius. However, the relaxation of the molten regions in the three targets is very different: nearly perfect recrystallization in MgO recovers the damage to an almost virgin state whereas only partial recovery in Al_2_O_3_ is observed and almost no recovery is seen in YAG. We demonstrated a correlation between the lattice structure and track crystallization efficiency. Simulations enabled us to identify that the kinetics of recrystallization of oxygen atoms is faster at the interface between the melted material and epitaxially restoring crystalline structure in tracks in MgO and Al_2_O_3_.

## Supplementary information

CDF reconstruction of YAG and Cross-sectional MD images

## Data Availability

All data generated and analyzed in this study are available upon request to the authors.
