# Supplementary material for: Recrystallization as the governing mechanism of ion track formation
Source: Sci Rep. 2019 Mar 7;9:3837. doi: 10.1038/s41598-019-40239-9 (PMC6405954; doi:10.1038/s41598-019-40239-9)
Supplement: Supplementary file 1 — CDF reconstruction of YAG and Cross-sectional MD images [file 41598_2019_40239_MOESM1_ESM.pdf]

Supplementary Materials

for

Recrystallization as the governing mechanism of ion track formation

R.A. Rymzhanov[1,2,3,*], N. Medvedev[4,5], J.H. O'Connell[6], A. Janse van Vuuren[6], V.A. Skuratov[1,7,8], A.E. Volkov[1,9,10,11]

[1] *Joint Institute for Nuclear Research, Joliot-Curie 6, 141980 Dubna, Moscow Region, Russia;*
[2] *The Institute of Nuclear Physics, Ibragimov St. 1, 050032 Almaty, Kazakhstan;*
[3] *L.N. Gumilyov Eurasian National University, Satpayev St. 2, 010008 Astana, Kazakhstan;*
[4] *Institute of Physics, Czech Academy of Sciences, Na Slovance 2, 182 21 Prague 8, Czech Republic;*
[5] *Institute of Plasma Physics, Czech Academy of Sciences, Za Slovankou 3, 182 00 Prague 8, Czech Republic;*
[6] *Nelson Mandela University, University way, Summerstrand, 6001 Port Elizabeth, South Africa*
[7] *National Research Nuclear University MEPhI, Kashirskoye sh., 31, 115409 Moscow, Russia;*
[8] *Dubna State University, Universitetskay 19, 141980 Dubna, Moscow Region, Russia;*
[9] *National Research Center 'Kurchatov Institute', Kurchatov Sq. 1, 123182 Moscow, Russia;*
[10] *Lebedev Physical Institute of the Russian Academy of Sciences, Leninskij pr., 53,119991 Moscow, Russia.*
[11] *National University of Science and Technology MISiS, Leninskij pr., 4,119049 Moscow, Russia*

*rymzhanov@jinr.ru

1. CDF reconstruction of YAG

Within our Monte Carlo (MC) approach TREKIS, we apply the dynamic structure factor - complex dielectric function (DSF-CDF) formalism [1,2] to obtain reliable cross sections of interactions of a charged particle with matter. The formalism arises within the first Born approximation, when the kinetic energy of a projectile is much larger than the potential energy of its interaction [3]. This formalism takes automatically into account collective modes of target response resulting from spatial and temporal correlations in the electronic and atomic ensembles.

For scattering of charged particles, the DSF can be expressed in terms of the loss function of a target (an imaginary part of the complex dielectric function, $\varepsilon(\omega,q)$) resulting in the following form of the cross section in a homogeneous isotropic system [4–6]:

$$\frac{d^2\sigma}{d(\hbar\omega)d(\hbar q)} = \frac{2\left(Z_e(v)e\right)^2}{n_{sc}\pi\hbar^2 v^2}\frac{1}{\hbar q}\left[1-e^{-\frac{\hbar\omega}{k_B T}}\right]^{-1}\mathrm{Im}\left(\frac{-1}{\varepsilon(\omega,q)}\right) \qquad (1)$$

where $\hbar\omega$ is the transferred energy and $\hbar q$ is the transferred momentum ($\hbar$ is the Planck's constant); Z_e is the effective charge of the incident particle [7]; e is the electron charge; v is the velocity of the particle; n_{sc} is the density of scattering centers: $n_{sc}= n_{at}$, the atomic density for scatterings on the

lattice, or $n_{sc}= n_{el}$, the density of electrons for scatterings on the electronic system; T is the temperature of the sample (room temperature in our case) and k_B is the Boltzmann constant.

Ritchie and Howie algorithm for artificial oscillators in the dipole approximation is employed to restore the CDF from the experimental optical data on photon scattering [2]:

$$\mathrm{Im}\left[\frac{-1}{\varepsilon(\omega, q=0)}\right] = \sum_{i=1}^{N^{os}} \frac{A_i \gamma_i \hbar\omega}{[\hbar^2\omega^2 - E_{0i}^2(q=0)]^2 + (\gamma_i \hbar\omega)^2}, \quad (2)$$

here, E_{0i} means the characteristic energy of the i-th oscillator, A_i is the fraction of electrons with energy E_{0i}, and γ_i is the i-th energy damping coefficient [8].

We used experimentally measured optical constants in studied materials to define the coefficients in Eq.(2). The data and approximation coefficients for alumina and magnesia can be found in our previous works [7] and [9], respectively. For YAG, the optical constants for low-energy photons (<30 eV) are taken from [10], while the data for high-energy photons are from [11]. These data are shown in **Figure 1**. The extracted coefficients for Eq.(2) are given in **Table 1**.

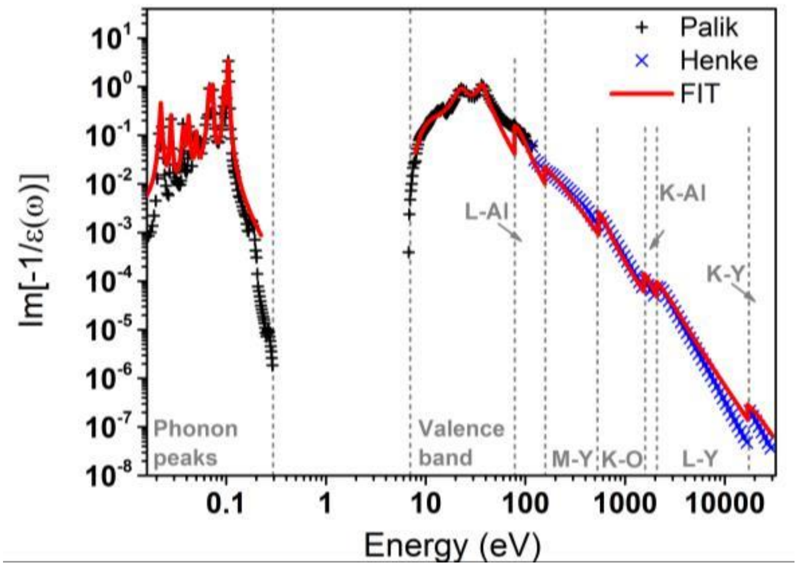

Figure 1. The experimental (Palik [10]) and calculated (Henke [11]) as well as approximated inverse imaginary part of CDF in $Y_3Al_5O_{12}$. Vertical dashed lines mark the different atomic levels in different elements according to the ionization potentials.

Table 1 The coefficients of the complex dielectric function in $Y_3Al_5O_{12}$ in the form of oscillator functions approximating the experimental optical coefficients: the total ps-sum gives 0.934 (6.6 % deviation from 1).

Name	E_{0i}	A_i	γ_i	*f*-sum (Number of electrons)
Valence Band	7	-4.05	5	113.74 (114)
	12	10	10	
	23	115	8	
	37	445	27	
	37	160	7	
L-shell of Al	80	364	40	40.03 (40)
M-shell of Y	157	485	250	60.36 (60)
K-shell of O	540	236	250	24.04 (24)
K-shell of Al	1540	95	750	9.94 (10)
L-shell of Y	2100	205	2000	23.92 (24)
K-shell of Y	17000	50	20000	5.96 (6)
Phonons	0.022	1.00E-05	1.00E-03	--
	0.028	7.00E-06	1.00E-03	
	0.037	1.00E-05	2.00E-03	
	0.042	2.00E-05	2.00E-03	
	0.05	1.00E-05	2.00E-03	
	0.068	2.70E-04	4.00E-03	
	0.073	1.40E-04	2.00E-03	
	0.095	3.50E-04	4.00E-03	
	0.1045	1.20E-03	3.00E-03	
Total:				277.99 (278)

Using the derived coefficients, the calculated energy losses of Xe ion in YAG are presented in **Figure 2**. The results of SRIM [12] code are also demonstrated for comparison. An overall good agreement confirms an applicability of the obtained coefficients of the loss-function for modeling of SHIs impacts in $Y_3Al_5O_{12}$.

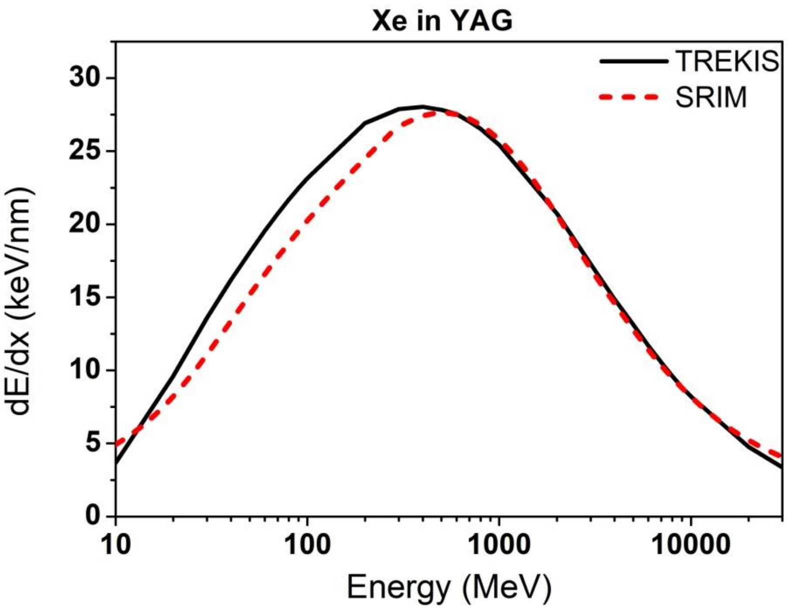

Figure 2 The calculated ion energy losses of Xe ion in YAG in comparison with SRIM code [12].

2. Cross-sectional MD images

Figure 3 shows cross sectional images of MD supercell of MgO, Al_2O_3 and YAG after an impact of 167 MeV Xe ion. The figure clearly demonstrates that only a small number of point defects are present in case of MgO; the crystalline and discontinuous ion track forms in Al_2O_3, while the damaged region in YAG is continuous and cylindrical.

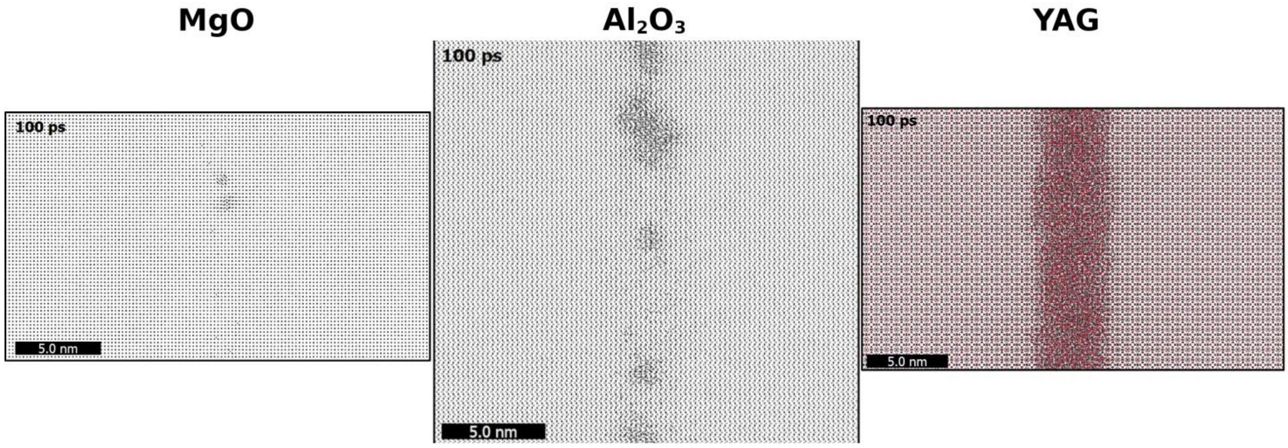

Figure 3 Modeled snapshots of 167 MeV Xe tracks in three materials at 100 ps after the ion passage: projection on (X,Z) plane, along the ion trajectory.

References

[1] L. Van Hove, Phys. Rev. **95**, 249 (1954).

[2] R. H. Ritchie and A. Howie, Philos. Mag. **36**, 463 (1977).

[3] L. D. Landau and L. M. Lifshitz, *Quantum Mechanics, Third Edition: Non-Relativistic Theory*, 3 edition, (Butterworth-Heinemann;, 1976).

[4] R. Kubo, Reports Prog. Phys. **29**, 255 (1966).

[5] A. M. Miterev, Physics-Uspekhi **45**, 1019 (2002).

[6] D. Pines, *Elementary Excitations in Solids* (W.A.Benjamin inc., New-York – Amsterdam, 1963).

[7] N. A. Medvedev, R. A. Rymzhanov, and A. E. Volkov, J. Phys. D. Appl. Phys. **48**, 355303 (2015).

[8] R. A. Rymzhanov, N. A. Medvedev, and A. E. Volkov, Nucl. Instruments Methods Phys. Res. Sect. B Beam Interact. with Mater. Atoms **388**, 41 (2016).

[9] R. A. Voronkov, R. A. Rymzhanov, N. A. Medvedev, and A. E. Volkov, Nucl. Instruments Methods Phys. Res. Sect. B Beam Interact. with Mater. Atoms **365**, 468 (2015).

[10] E. D. Palik, *Handbook of Optical Constants of Solids* (Academic Press, San Diego, 1985).

[11] B. L. Henke, E. M. Gullikson, and J. C. Davis, At. Data Nucl. Data Tables **54**, 181 (1993).

[12] J. P. Ziegler, U. Biersack, and J. F. Littmark, *The Stopping and Range of Ions in Solids* (Pergamon Press, New York, 1985).
